# Associations between daily dietary carbohydrate intake and TIR in adults with type 1 diabetes

**DOI:** 10.3389/fnut.2025.1638849

**Published:** 2025-09-03

**Authors:** Yunying Cai, Xi Li, Xin Xiong, Lun Zhang, Jianfeng He, Heng Su

**Affiliations:** ^1^The Endocrinology Department, First People's Hospital of Yunnan Province, The Affiliated Hospital of Kunming University of Science and Technology, Type 1 Diabetes Alliance of Yunnan Province, Kunming, China; ^2^Medical School, Kunming University of Science and Technology, Kunming, China; ^3^Faculty of Information Engineering and Automation, Kunming University of Science and Technology, Kunming, China; ^4^The Clinical Nutrition Department, First People's Hospital of Yunnan Province, The Affiliated Hospital of Kunming University of Science and Technology, Kunming, China

**Keywords:** continuous glucose monitoring, diet effect, carbohydrate, type 1 diabetes, hypoglycemia

## Abstract

**Objective:**

To assess the association between daily carbohydrate (CHO) intake and glycemic control in adults with type 1 diabetes (T1D).

**Methods:**

Patients with T1D who received continuous glucose monitoring (CGM) to manage their blood glucose levels were enrolled in the study. A dietitian analyzed dietary components, including carbohydrate, protein, and fat percentages in the total dietary intake. Mean individual daily CHO intake (MIDC) and relative deviation from MIDC (< 80% low; 81%−120% medium, >120% high CHO consumption) were compared with parameters of glycemic control assessed by CGM.

**Results:**

Records from 36 patients [11 male, 25 female; age 39.5 ± 13.9 years; HbA1c 9.0 ± 2.8% (75 ±31 mmol/mol)]. Provided 356 days of data for a total of 1,068 meals. Time in range (3.9–10 mmol/l) for low, medium, and high CHO consumption was 81.6 (70.96, 90.28)%, 74.65 (59.55, 84.9)%, and 64.58 (51.04, 77.78)%, respectively (*P* < 0.001). Time above range (>10 mmol/L) was 9.55 (1.39, 17.95)%, 10.42 (2.78, 27.43)%, and 27.08 (11.46, 47.92)%, respectively (*P* < 0.001). There was no between-group difference for time in hypoglycemia (< 3.9 mmol/L; *P* = 0.136). After adjusting for HbA1c, total calorie intake, and total daily insulin dose, carbohydrate intake was negatively correlated with achieving TIR ≥ 70%.

**Conclusions:**

Daily CHO intake was inversely associated with glycemic control in adults with T1D. A carbohydrate energy percentage between 40% and 50% and a relatively low daily carbohydrate intake may be a strategy to optimize glucose control in suboptimal-controlled T1D in real-world settings.

## Introduction

Type 1 diabetes is a metabolic disorder characterized by the progressive autoimmune destruction of pancreatic β-cells in genetically predisposed individuals, ultimately leading to absolute insulin deficiency. According to the International Diabetes Federation (IDF), in 2022, an estimated 8.75 million individuals worldwide were living with T1D (95% confidence interval: 8.4–9.1 million) (1). Despite geographical variations, the overall annual incidence of T1D is projected to increase by ~3%−4% (1– 4).

Despite the significant advancements in insulin therapy that have markedly enhanced patient outcomes, glycemic control in Type 1 Diabetes (T1D) continues to be suboptimal. For instance, the T1D Exchange registry indicates that only 21% of adult patients attain a hemoglobin A1c (HbA1c) level below 7.0% (5). Inadequate long-term glycemic control can precipitate severe acute and chronic complications. Epidemiological studies conducted in the United States and Europe over the past decade have emphasized a concerning increase in hyperglycemic emergencies among adults with T1D (6, 7). Additionally, the overall mortality rate resulting from diabetic ketoacidosis (DKA) presents a significant concern; data from national registries in Scotland reveal that nearly 16% of fatalities in T1D patients under the age of 50 are attributable to diabetes-related coma or DKA (8). Chronic microvascular complications, such as retinopathy, nephropathy, and neuropathy, demonstrate occurrence rates as high as 30%−50% among T1D patients with a disease duration exceeding 10–15 years (9, 10).

Another area of concern is the cardiovascular disease (CVD) risk associated with T1D. The ESC CVD risk stratification applied to 34,705 T1D subjects in the Italian AMD Annals Initiative found that 64.7% of these individuals were at very high risk for CVD. Furthermore, females diagnosed with T1D before the age of 10 experienced a loss of 17.7 life years (95% CI: 14.5–20.4), while males lost 14.2 life years (95% CI: 12.1–18.2) (10).

Postprandial glucose fluctuations significantly challenge glycemic control in individuals with T1D. In patients with reasonable diabetes management (HbA1c < 7.3%), postprandial plasma glucose contributes 70% to overall glycemic variability (11). CHO intake is recognized as a primary determinant of postprandial glucose levels and glucose variability (12, 20). The ISPAD 2022 guidelines recommend that carbohydrates account for 40%−50% of total energy intake (13). However, the 2025 guidelines from the American Diabetes Association (ADA) emphasize that there is no ideal macronutrient proportion for individuals with diabetes (14). The impact of daily carbohydrate intake on glycemic control and fluctuations remains unclear. In this pilot study, we aim to investigate the relationship between dietary CHO intake and glucose levels, as monitored via CGM, in free-living conditions among individuals with T1D. We hypothesize that dietary carbohydrate intake is the primary driver of daily time in range (TIR).

## Methods

### Participants

This observational study, conducted at the Endocrinology Department of Yunnan Provincial First People's Hospital, enrolled participants with type 1 diabetes (T1D) from June 2023 to June 2024. Eligible participants met the ADA diagnostic criteria for T1D, had a disease duration of at least six months, were aged between 18 and 55, and had HbA1c levels ranging from 6.5% to 11.0% in 3 months before enrollment. Participants needed to follow a basal-bolus insulin regimen using multiple daily injections (MDI) or continuous subcutaneous insulin infusion (CSII), and they were required to maintain a dietary record independently. Exclusion criteria included refusal to utilize continuous glucose monitoring (CGM) technology or share blood glucose data; usage of automated insulin delivery systems; any occurrence of diabetic ketoacidosis (DKA) or severe complications, such as eGFR < 30 mL/min/1.73 m^2^ or proliferative retinopathy, within 3 months before enrollment; active gastrointestinal disorders or celiac disease; and a history of cognitive dysfunction or mental illness. This study was approved by the Medical Ethics Committee of Yunnan Provincial First People's Hospital (Approval No: KHLL2022-KY165), and all participants provided written informed consent.

### Nutritional assessment

Nutritional intake was evaluated using a standardized food weighing method along with dietary diaries. The research center provided electronic kitchen scales (accuracy ± 1 g) and standardized dietary record booklets. Nutritional data for packaged foods were obtained from food labels, while data for non-packaged foods were analyzed with the TangTangQuan dietary analysis software (Aibaowei Biotechnology, China). Before enrollment, participants underwent a 30-min standardized training session conducted by a registered dietitian, which covered food portion estimation, weighing techniques, and addressing exceptional circumstances (e.g., semi-quantitative recording for meals eaten out).

The dietary diary needed to capture the following details: food type and brand, time of consumption, dining context (whether at home or dining out), and insulin dosage. Records were omitted from the final analysis if the daily energy intake fell below 800 kcal or meals were missed. The same dietitian evaluated all dietary diaries, determining daily caloric intake and the amounts of carbohydrates, fats, and proteins. A minimum of 3 days of dietary records was necessary, with an average daily caloric intake surpassing 800 kcal; records lacking complete meal entries were excluded from the final analysis. All dietary records were analyzed with TangTangQuan^®^ software to calculate total caloric intake and the macronutrient breakdown. Mean individual daily carbohydrate intake (MIDC, g/day) and the rMIDC (relative deviation from MIDC, %MIDC) were utilized to reflect variations in daily carbohydrate consumption between individuals. Based on rMIDC, participants were stratified into low ( ≤ 80%), medium (81%−120%), and high (≥120%) (15), e.g., 80% rMIDC: mean individual daily CHO intake ^*^ 0.8.

### Demographic and biomedical data

The following parameters were collected: demographics (age, sex, height, weight, duration of diabetes); metabolic indicators (HbA1c, liver and kidney function, lipid profile including total cholesterol, high-density lipoprotein cholesterol, low-density lipoprotein cholesterol, and triglycerides); and treatment information [type of insulin therapy (MDI or CSII), average daily total insulin dose (U/kg/d) from the past week, and understanding of carbohydrate counting (yes/no)].

### Glycemic outcomes

For analysis, the following CGM metrics were evaluated: percentage of time in the glucose range of 3.9–10 mmol/L (%TIR), percentage of time below range < 3.9 mmol/L (%TBR), and percentage of time above range >10 mmol/L (%TAR), mean glucose levels, and glycemic variability calculated as the coefficient of variation (CV = SD/mean).

### Statistical analysis

All statistical analyses were performed using R software (version 4.2.2; R Foundation for Statistical Computing, Vienna, Austria). Data preprocessing and cleaning involved several R packages: magrittr (version 2.0.3) for pipeline operations, dplyr (version 1.1.1) and data.table (version 1.14.8) for data manipulation, tidyr (version 1.3.0) for data tidying, and stringr (version 1.5.0) for string processing. Missing values were handled using the drop_na() function from the tidyr package.

Depending on the circumstances, categorical variables were compared using Pearson's chi-square test or Fisher's exact test. Continuous variables were assessed with the independent samples *t*-test if they were normally distributed; otherwise, the Mann-Whitney *U* test was employed, with normality established via the Shapiro-Wilk test. Generalized linear mixed models were implemented using the lme4 package (version 1.1-32). In the model, total CHO intake was treated as a fixed effect, while HbA1c, daily insulin intake, and total caloric intake were included as covariates. The sample ID was designated as a random effect. Forest plots were generated using the forestplot package (version 3.1-1).

## Results

### Participant characteristics

A total of 36 patients with T1D were included in the study, consisting of 25 females (69.4%) and 11 males (30.6%)—thirteen participants (36.1%) utilized carbohydrate counting. We collected a comprehensive dietary record spanning 356 days, which included 1,068 meals, a median recording duration of 9 days. The participants had a mean age of 39.5 ± 13.9 years and a diabetes duration of 13.2 ± 9.2 years. The mean glycated hemoglobin (HbA1c) level was 9.0% ± 2.8%, and the average total daily insulin dosage was 34.7 ± 11.0 units. The mean time in range (TIR) was also 64.2 ± 12.1% (Table 1).

**Table 1:** Baseline characteristics.

**Characteristics**	**Mean ±SD or Median (IQR)**
**Participants, *n***	**36**
Female, *n* (%)	25 (69.4)
Age, years	39.5 ± 13.9
BMI, kg/m^2^	21.6 ± 3.2
Duration of diabetes, years	13.2 ± 9.2
Portion of using carbohydrate counting	36.1%
HbA1c % mmol/mol	9.0 ± 2.8
	75 ± 31
Fasting C-Peptide (nmol/L)	0.08 ± 0.01
MDI therapy, *n* (%)	28 (77.8)
Pump therapy, *n* (%)	8 (22.2)
TDD (U)	34.7 ± 11.0
TDD/Weight [U/(kg.day)]	0.66 ± 0.27
Mean daily basal insulin (U)	13.7 ± 5.5
Mean daily bolus insulin (U)	19.46 ± 1.30
TIR (%)	64.2 ± 12.1

Data are mean ± SD or median (IQR), unless otherwise indicated.

BMI, body mass index; MDI, multiple daily insulin injections; TDD, Total insulin delivered; TIR, time in range.

### Daily carbohydrate intake linked to glycemic outcomes

Table 2 summarizes daily CHO intake, total daily insulin dosage, and CGM metrics categorized by rMIDC. The mean daily CHO intake was 164.21 ± 60 g. Specifically, daily CHO intake for the low, medium, and high rMIDC groups was 108.3 g (95.33, 120.2), 152.8 g (142.4, 172.75), and 251.7 g (222.2, 269.25), respectively (*P* < 0.001). The total calorie intake for the low, medium, and high rMIDC groups was 1,012.2 Kcal (889.47, 1,186.76), 1,277.7 Kcal (1,101.5, 1,471), and 1,748.8 Kcal (1,608.86, 1,903.8), respectively (*P* < 0.001). CHO intake accounted for 42.09% ± 9.04%, 49.65% ± 9.72%, and 58.17% (52.73, 63.71%) of total daily energy in the low, medium, and high rMIDC groups, respectively (*P* < 0.001).

**Table 2:** Associations between carbohydrate intake and blood glucose control.

**VariableCGM metrics**	**rMIDC ≤ 80% (days = 120)**	**rMIDC 80%−120% (days = 147)**	**rMIDC ≥120%(days = 89)**	***P* value**
TIR (3.9–10 mmol/L) (%)	81.6 (70.96, 90.28)	74.65 (59.55, 84.9)^***^	64.58 (51.04, 77.78)^***^###	< 0.001
TBR (< 3.9 mmol/L) (%)	4.46 (0.69, 11.8)	6.6 (1.56, 15.28)^*^	3.43 (0, 13.19)^##^	0.136
TAR (>10 mmol/L) (%)	9.55 (1.39, 17.95)	10.42 (2.78, 27.43)	27.08 (11.46, 47.92)^***^###	< 0.001
TIR (3.9–10 mmol/L) ≥ 70% (%)	80 (96, 120)	58.5 (86, 147)^***^	40.45 (36, 89)^***^#	< 0.001
TIR ≥ 70% & TBR < 5% (%)	44.17 (53, 120)	22.45 (33, 147)^***^	19.1 (17, 89)^***^	< 0.001
TIR ≥ 70% & TBR < 5% & TAR < 25% (%)	39.17 (47, 120)	21.77 (32, 147)^**^	14.61 (13, 89)^***^	< 0.001
Mean glucose (mmol/L)	6.93 (6.27, 7.92)	6.97 (6.13, 8.39)	8.27 (7.1, 10.03)^***^###	< 0.001
SD (mmol/L)	1.97 (1.71, 2.38)	2.23 (1.9, 2.93)^***^	2.7 (2.02, 3.33)^***^#	< 0.001
MAGE (mmol/L)	5.03 (4.12, 6.78)	6.08 (4.78, 7.93)^**^	6.82 (5.22, 8.81)^***^#	< 0.001
LAGE (mmol/L)	8.6 (7.27, 10.5)	10 (8.05, 12.15)^**^	11.03 (6.82, 15.26)^***^	< 0.001
CV glucose (%)	28.45 (23.93, 33.99)	32.02 (27.64, 38.77)^***^	33.71 (21.9, 45.52)^**^	< 0.001
**Insulin dosage**				
TDD (U)	30 (22, 37)	39 (29.95, 43)^***^	36 (29, 39)^**^##	0.173
Basal insulin dosage (U)	12.3 (9.5, 18.3)	13 (12.3, 14.95)^*^	13 (12, 14.25)	0.084
Bolus insulin dosage (U)	15.75 (12.2, 20)	25 (16, 28)^***^	21 (16.8, 25)^***^##	0.045

Asterisks denote significance levels of postestimations comparing the rMIDC group < 80% against the rMIDC group 80%−120%, and the rMIDC group ≥ 120%: ^*^P < 0.05, ^**^P < 0.01, ^***^P < 0.001. ^#^ denote significance levels of postestimations comparing the rMIDC group 80%−120%, and against the rMIDC group ≥ 120%: ^#^*P* < 0.05, ^##^*P* < 0.01, ^###^*P* < 0.001.

rMIDC, the relative deviation from mean individual daily carbohydrate intake; TIR, time in range; TAR, time above range; TBR, time below range; SD, standard deviation; MAGE, mean amplitude of glycemic excursions; LAGE, largest amplitude of glycemic excursions; CV, coefficient of variation; TDD, total insulin delivered.

TIR values for the low, medium, and high rMIDC groups were 81.6% (70.96, 90.28), 74.65% (59.55, 84.9), and 64.58% (51.04, 77.78) (*P* < 0.001). TAR percentages were 9.55% (1.39, 17.95), 10.42% (2.78, 27.43), and 27.08% (11.46, 47.92) (*P* < 0.001). No significant difference in TBR was observed (*P* = 0.136). The proportion of subjects achieving comprehensive control, defined as TIR ≥ 70%, TBR < 5%, and TAR < 25%, was 39.17%, 21.77%, and 14.61% in the low, medium, and high rMIDC groups, respectively (*P* < 0.001).

Additionally, glycemic variability metrics, including the coefficient of variation (CV), mean amplitude of glycemic excursions (MAGE), and largest amplitude of glycemic excursions (LAGE), showed significant differences among the three groups (*P* < 0.001). Compared to the medium and high rMIDC groups, the low rMIDC group exhibited decreased diurnal blood glucose variability. Concerning insulin dosage, significant differences were noted only in bolus insulin dosage across the groups (*P* < 0.001). Compared to the medium and high rMIDC groups, the low rMIDC group observed lower bolus insulin dosage.

### Predictor of TIR ≥ 70%

**Figure 1:**
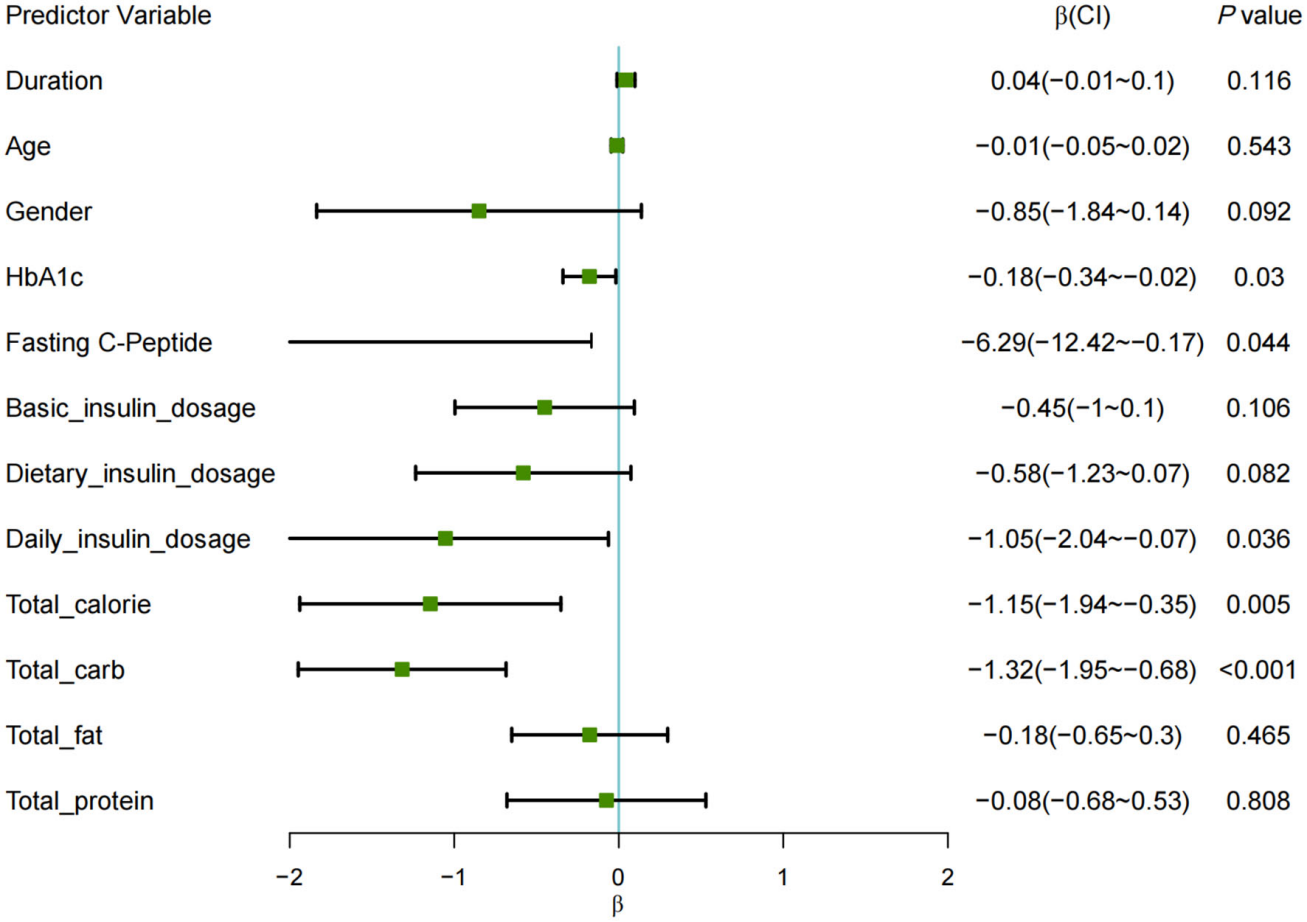
Forest plot of restricted mixed-effects model for TIR ≥ 70%. Each square represents the point estimate (β), with horizontal lines denoting 95% CIs. The range of the X-axis is set from −2 to 2, with any lines that extend beyond the axis indicating confidence interval (CI) values that fall outside this range.

The forest plot analysis (Figure 1) displays the effect sizes (β coefficients) and their corresponding 95% confidence intervals (CIs) for the categorical predictor defined as TIR ≥ 70%.

The results indicated several factors negatively associated with TIR: HbA1c showed a significant effect (β = −0.18, *P* < 0.05); fasting C-peptide levels (β = −6.29, *P* < 0.05); total daily insulin dose (β = −1.05, *P* < 0.05); total daily caloric intake (β = −1.15, *P* < 0.01); and daily carbohydrate intake (β = −1.32, *P* < 0.001), all demonstrating a negative impact on the likelihood of achieving TIR ≥ 70%.

After adjusting for HbA1c, total calorie intake, and total daily insulin dose, carbohydrate intake was negatively correlated with achieving TIR ≥ 70% [β = −1.19, OR = 0.30; 95% CI (0.12, 0.80); *P* = 0.016]. Specifically, a 50% increase in CHO intake was associated with a 49% decrease in the probability of meeting the TIR target.

Each square represents the point estimate (β), with horizontal lines denoting 95% CIs. The range of the X-axis is set from −2 to 2, with any lines that extend beyond the axis indicating confidence interval (CI) values that fall outside this range.

## Discussion

In this study, we investigated the effects of dietary CHO intake on glucose levels and glycemic control in adults with T1D and high baseline HbA1c who lived in free-living conditions. Our findings indicate a negative correlation between daily CHO intake and glycemic control. However, the benefits of CHO restriction may differ in individuals with well-controlled T1D.

Previous research has primarily focused on the relationship between macronutrient distribution and HbA1c levels. For instance, a study involving 136 adolescents with T1D (aged 8–17 years) over a one-year observation period found that greater CHO intake was associated with lower HbA1c and higher CV (16). However, HbA1c reflects average glucose levels over the preceding 3–4 months, and fluctuations in macronutrient intake during this period may compromise the reliability of this association. Cherubini et al. observed in a cohort of 197 children with T1D that a diet comprising 40%−44% carbohydrates was associated with a significantly higher percentage of participants achieving TIR > 70% compared to those consuming 45%−50% carbohydrates (17). Similarly, Lehmann et al. (15) noted in a study of 36 adults with T1D using a Hybrid Closed-Loop System that daily CHO intake inversely correlated with glycemic control, particularly in patients who frequently used the automatic mode. Our study corroborates these findings, as lower CHO intake significantly improved TIR by 17.02% compared to higher CHO intake. Additionally, parallel to previous studies identifying carbohydrate intake as a negative predictor of TIR during breakfast and dinner (18), our research found that CHO intake was also a negative predictor of 24-h TIR.

While HbA1c explains only a portion of the risk for diabetes-related complications, recent studies have shown that glycemic variability is associated with various microvascular and macrovascular complications of diabetes (19). Previous research has indicated that CHO intake is linked to greater postprandial glycemic variability over 3–5 h (12, 20). Data from the Type 1 Diabetes Exercise Initiative Pediatric (T1DEXIP) study revealed that higher CHO meals increased postprandial variability in glucose CV and SD (20). However, the relationship between CHO intake and daily blood glucose fluctuations has yet to be validated (21). Our study found that CHO intake is associated with higher 24-h SD, MAGE, and LAGE. Compared to the rMIDC group < 80% (28.45%), the rMIDC groups 80%−120% (32.02%) and ≥120% (33.71%) exhibited increased CV. Consistent with our findings, de Wit et al. observed that higher CHO intake correlated with increased CV in a cohort of 470 T1D patients monitored with continuous glucose monitoring (CGM) over 2 weeks using three-day dietary diaries [OR for CV < 25% = 0.69 (95% CI 0.51, 0.90)] (22). Our study further demonstrated that fluctuations in daily CHO intake are positively associated with CV beyond its relationship with HbA1c (23). This supports the potential benefits of maintaining consistency in CHO intake routines, as excessive dietary flexibility may hinder glycemic management.

Regarding hypoglycemia risk, our study found no significant impact of varying CHO intake on the 24-h TBR. The effects of low-CHO diets on hypoglycemia risk in T1D remain debated. For example, a study of 1,040 children with T1D found that those on a low-CHO diet—defined as deriving less than 26% of energy from carbohydrates—experienced more time in hypoglycemia (< 3.9 mmol/L) compared to those following a normal diet (8% vs. 5%) (24). In contrast, Lennerz et al. (25) conducted an online survey assessing the impact of very low carbohydrate diets (VLCD) on T1D in children and adults. Participants adhering to a regimen of daily CHO intake of 36 ± 15 grams experienced a hypoglycemia incidence of only 1% over 2.2 ± 3.9 years. Furthermore, a study involving 285 adult T1D patients, with CHO intake ranging from 31.2 ± 6.9% to 56.5 ± 6.8% of total daily energy, revealed that those in the lowest quintile (Q1) reported experiencing severe hypoglycemia less frequently compared to those in the third quintile (Q3) (Q1: 60.0% vs. Q3: 31.0%). However, there were no differences in the frequency of grade 2 hypoglycemic events across quartiles (26). Unlike previous studies on low-CHO diets, which associate low intake with an increased risk of hypoglycemia, we conclude that a CHO intake comprising 40%−50% of total energy does not elevate the risk of hypoglycemia. Otherwise, we found that daily CHO intake was negatively correlated with achieving a TIR ≥ 70% (OR = 0.30), indicating that a 50% increase in daily CHO consumption is associated with a 49% decrease in the probability of achieving a TIR ≥ 70%. Maintaining daily carbohydrate energy contributions at 40%−50% enables moderate CHO intake reduction to significantly increase TIR without elevating hypoglycemia risk.

We acknowledge several limitations in this study. First, the single-center observational design restricts our ability to eliminate selection bias or other systematic errors, such as regional dietary habits, which may limit the applicability of our results. Additionally, the limited observation period necessitates the evaluation of longer-term effects. Second, our data do not account for the type of carbohydrates consumed. At the same time, quality factors (e.g., glycemic index and fiber content) and other variables (such as exercise, meal frequency, stress, and sleep) may also influence glucose dynamics. Future research should aim to disentangle the effects of CHO quantity from CHO quality, as a diet with the same carbohydrate amount but higher fiber content may yield different glycemic outcomes. Lastly, the participants primarily used empirical estimations for pre-meal insulin dosages rather than standardized carbohydrate counting methods for insulin dosing.

## Conclusion

Our study provides real-world evidence that for adults with suboptimally controlled T1D, aiming for a carbohydrate intake of 40%−50% of total energy and, crucially, maintaining consistency in daily intake, is a safe and effective strategy to increase TIR and reduce glycemic variability without elevating the risk of hypoglycemia.

## Data Availability

The raw data supporting the conclusions of this article will be made available by the authors, without undue reservation.
